# Cellular basis of neuroepithelial bending during mouse spinal neural tube closure

**DOI:** 10.1016/j.ydbio.2015.06.003

**Published:** 2015-08-15

**Authors:** Suzanne G. McShane, Matteo A. Molè, Dawn Savery, Nicholas D. E Greene, Patrick P.L. Tam, Andrew J. Copp

**Affiliations:** a Newlife Birth Defects Research Centre, Institute of Child Health, University College London, 30 Guilford Street, London WC1N 1EH, UK; b Embryology Unit, Children’s Medical Research Institute and Sydney Medical School, University of Sydney, Westmead, New South Wales 2145, Australia

**Keywords:** Mouse, Embryo, Neurulation, Neural tube closure, Cell proliferation

## Abstract

Bending of the neural plate at paired dorsolateral hinge points (DLHPs) is required for neural tube closure in the spinal region of the mouse embryo. As a step towards understanding the morphogenetic mechanism of DLHP development, we examined variations in neural plate cellular architecture and proliferation during closure. Neuroepithelial cells within the median hinge point (MHP) contain nuclei that are mainly basally located and undergo relatively slow proliferation, with a 7 h cell cycle length. In contrast, cells in the dorsolateral neuroepithelium, including the DLHP, exhibit nuclei distributed throughout the apico-basal axis and undergo rapid proliferation, with a 4 h cell cycle length. As the neural folds elevate, cell numbers increase to a greater extent in the dorsolateral neural plate that contacts the surface ectoderm, compared with the more ventromedial neural plate where cells contact paraxial mesoderm and notochord. This marked increase in dorsolateral cell number cannot be accounted for solely on the basis of enhanced cell proliferation in this region. We hypothesised that neuroepithelial cells may translocate in a ventral-to-dorsal direction as DLHP formation occurs, and this was confirmed by vital cell labelling in cultured embryos. The translocation of cells into the neural fold, together with its more rapid cell proliferation, leads to an increase in cell density dorsolaterally compared with the more ventromedial neural plate. These findings suggest a model in which DLHP formation may proceed through ‘buckling’ of the neuroepithelium at a dorso-ventral boundary marked by a change in cell-packing density.

## Highlights

• • The closing mouse spinal neural plate bends at the midline and dorsolaterally.
• • Basal nuclear localisation causes midline, but not dorsolateral, bending.
• • Neuroepithelial cells translocate into the dorsolateral neural plate during closure.
• • A discontinuity of cell density results between dorsal and ventral neural plate.
• • Buckling at this discontinuity may result in dorsolateral neural plate bending.

## Graphical abstract

**Figure:**
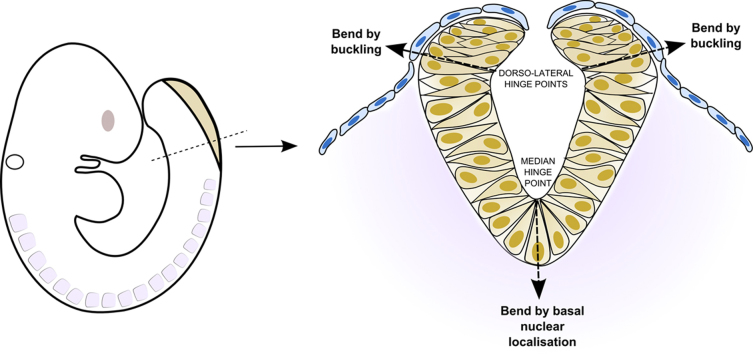

## 1 Introduction

Neurulation is the embryonic process by which the neuroepithelium bends and fuses dorsally to create the closed neural tube. A key morphogenetic component of neurulation is the stereotypical pattern of neuroepithelial bending by which the neural folds become elevated and apposed, enabling the adhesion and fusion that completes neural tube closure. In the spinal region of the mouse embryo, the neural plate bends acutely and focally at the midline (the median hinge point; MHP), creating the ‘neural groove’ with a V-shaped cross-section, and at paired dorsolateral hinge points (DLHPs) to generate longitudinal furrows that bring the neural fold tips towards each other in the dorsal midline. A switch from MHP to DLHP bending occurs as closure progresses rostro-caudally along the body axis (Shum and Copp, 1996; Ybot-Gonzalez and Copp, 1999). The *Zic2*-mutant mouse fails to develop DLHPs and subsequently exhibits extensive spina bifida (Elms et al., 2003; Ybot-Gonzalez et al., 2007), demonstrating the critical requirement for DLHP formation in the closure of the spinal neural tube.

The pattern of MHP and DLHP bending in the neural plate is regulated by mutually antagonistic dorsal and ventral embryonic signals. MHP bending is stimulated by notochordal factors including sonic hedgehog (Shh), whereas DLHP formation is simultaneously inhibited by Shh (Ybot-Gonzalez et al., 2002). Bone morphogenetic protein-2 (BMP2) secreted from the dorsal-most surface ectoderm also inhibits dorsolateral bending whereas Noggin, secreted from the tips of the neural folds, overcomes BMP2-mediated inhibition and enables DLHPs to form (Ybot-Gonzalez et al., 2007). In the upper spine, Shh is expressed strongly in the notochord and suppresses Noggin-mediated DLHP formation whereas, in the low spine, Shh production from the notochord is greatly diminished, Noggin is de-repressed, blocks BMP2 action, and DLHP formation occurs (Ybot-Gonzalez et al., 2007).

Contraction of apical actin microfilaments is conventionally considered essential for neural plate bending. However, mice with targeted defects of cytoskeletal genes – while often exhibiting cranial neural tube defects – rarely show closure defects of the trunk region (Copp, 2005). Moreover, cytochalasins that disassemble actin microfilaments disrupt cranial neural tube closure, whereas the process of spinal neural tube closure is cytochalasin-resistant and both MHP and DLHPs are formed properly (Ybot-Gonzalez and Copp, 1999). Hence, other mechanisms may be responsible for the bending of the neural plate in the spinal region.

Neuroepithelial shape change is a key factor in MHP formation. The pseudostratified neuroepithelium exhibits interkinetic nuclear migration, between basal and apical locations as the cell cycle progresses (Sauer, 1935; Langman et al., 1967). In chick and mouse embryos, the MHP is populated mostly by wedge-shaped cells with basally located, mainly S-phase, nuclei. In contrast, non-bending regions of the neural plate comprise a mixture of cells with wedge, spindle and inverted-wedge shapes (Schoenwolf and Franks, 1984; Smith et al., 1994). Hence, bending of the neural plate at the midline may be attributed to the preponderance of wedge-shaped cells due to the basal location of the interphase nuclei, as the cells spend longer in S-phase (Smith and Schoenwolf, 1987; Smith and Schoenwolf, 1988).

During chick neurulation, the DLHP comprises 55% wedge-shaped cells, compared with more than 70% in the MHP and fewer than 35% in non-bending neural plate (Schoenwolf and Franks, 1984). Neurulation in the mouse has not been subjected to such detailed cellular analysis, and it is unclear whether DLHP bending in the mouse neural plate is accomplished by a similar cellular wedging as at the MHP. Here, we found that DLHPs do not differ from non-bending neural plate in their content of cells with basal nuclei, arguing for a different mechanism of DLHP formation compared with the MHP. Analysis of cell proliferation during DLHP formation, combined with neuroepithelial vital cell labelling, reveals a net ventral-to-dorsal translocation of cells within the plane of the elevating neuroepithelium. Cells congregate in the dorsal neural fold and differential cell packing densities result, dorso-ventrally, within the neuroepithelium. A physical discontinuity dorso-ventrally may lead to neural plate bending specifically at the DLHP. These findings highlight a novel mechanism of epithelial morphogenesis during closure of the mammalian neural tube.

## 2 Materials and Methods

### 2.1 Mouse strains and embryo preparation

Mouse procedures were performed under the auspices of the UK Animals (Scientific Procedures) Act 1986 and ‘Responsibility in the Use of Animals for Medical Research’ (Medical Research Council, 1993). Mouse strains were: inbred CBA/Ca for morphometric analysis; random-bred CD1 mice for surface ectoderm removal; random-bred ARC/s and inbred BALB/c mice for live cell labelling. Embryonic day (E) 0.5 was noon on the day after overnight mating. Embryos were dissected in Dulbecco’s Modified Eagle’s Medium (DMEM) containing 10% fetal calf serum (FCS), staged by counting somites and rinsed in phosphate buffered saline (PBS) prior to fixation. For ^3^H-thymidine labelling, surface ectoderm removal, or live cell labelling, embryos were cultured whole, within yolk sac and amnion, as described (Cockroft, 1990). Surface ectoderm was removed surgically (Ybot-Gonzalez et al., 2002).

### 2.2 Morphometric analysis

Embryos were fixed for at least 24 h at 4 °C in 3% glutaraldehyde in 0.1 M sodium cacodylate buffer, pH 7.2, washed twice in distilled water and post-fixed in 1% aqueous osmium tetroxide for 10 min at room temperature, followed by 1-2 h at 4 °C. After two 10 min washes in distilled water, embryos were dehydrated through an ethanol series, followed by two 10 min changes of propylene oxide, then resin/propylene oxide (1:1), before embedding in Epon resin (Agar). Semi-thin transverse sections, 2.5 μm thick, were prepared using a glass knife on a Philips ultramicrotome. Sections were stained with 1% toluidine blue/ 1% borax, and mounted in DPX.

**Fig. 1:**
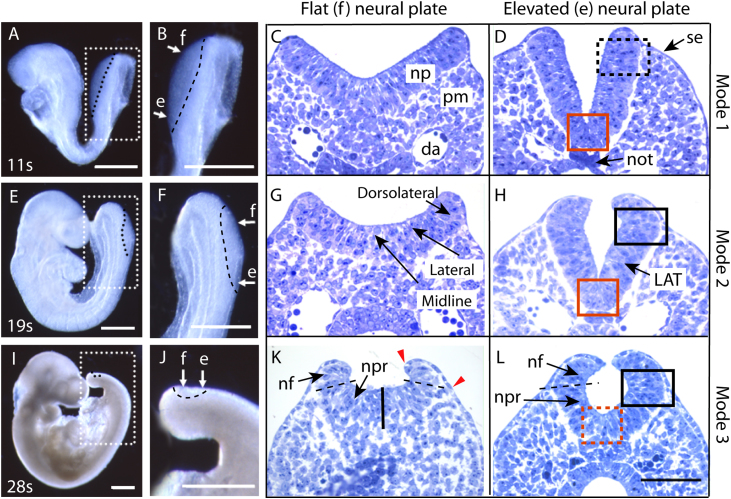
Embryo and posterior neuropore (PNP) morphology at successive stages of mouse spinal neurulation. (**A, B, E, F, I, J**) Whole embryos at E8.5 (A), E9.5 (E) and E10 (I) and enlarged views of the caudal region (B, F, J; white dotted boxes in A, E, I). The PNP (black dotted lines) shortens progressively as development proceeds. Arrows indicate level of sections through flat (f) and elevated (e) neural plate. (**C, D, G, H, K, L**) Transverse 2.5 μm thick plastic sections through the PNP showing transition from flat (C, G, K) to elevated (D, H, L) neural folds. At E8.5 (C, D; Mode 1), bending is solely at MHP (red box); at E9.5 (G, H; Mode 2), both MHP and DLHPs (black box) are present, with non-bending lateral neural plate (LAT) in between; at E10 (K, L; Mode 3), only DLHP bending occurs. Dorsolateral non-bending neural plate at Mode 1 (dotted black box, D) and midline non-bending neural plate at Mode 3 (dotted red box, L) were also analysed. Dashed lines (K, L): boundary between neural fold (nf) and neuroepithelium outside neural fold (npr); solid line (K): midline. Red arrowheads (K): surface ectoderm juxtaposed to neuroepithelium of the neural fold. Abbreviations: da, dorsal aorta; not, notochord; np, neural plate; pm, paraxial mesoderm; s, somite number. Scale bars: A,B,E,F,I,J: 0.5 mm; L (for all sections): 0.1 mm.

Embryos within specific somite number ranges were selected for analysis, in order to represent the characteristic morphology of the three Modes of neurulation: Mode 1 (MHP only), 10-12 somites; Mode 2 (MHP and DLHPs), 19-21 somites; Mode 3 (DLHPs only), 28-30 somites. Five embryos of each Mode were analysed with data gathered in each embryo from two rostro-caudal levels of the posterior neuropore (PNP): flat and elevated neural plate (Fig. 1).

**Fig. 2:**
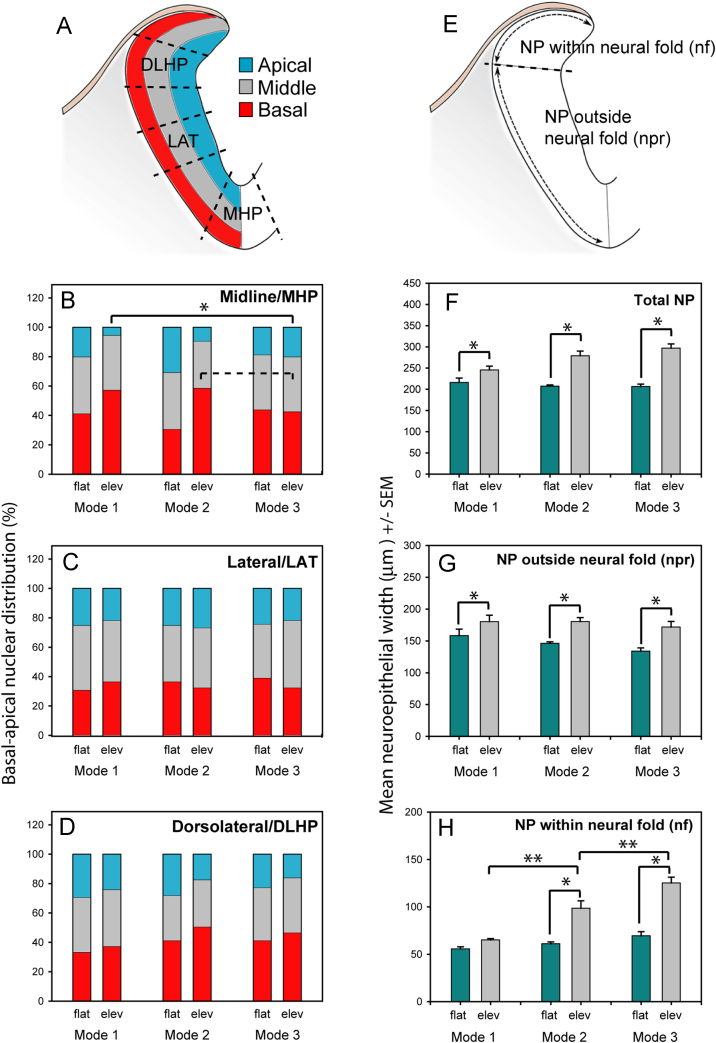
Nuclear position and neuroepithelial width during neural plate (NP) bending. (**A, E**) Diagrams illustrating measurements shown in graphs below. (**B-D**) Proportion of nuclei in basal (red), middle (grey) and apical (blue) thirds of the NP for midline (B; contains the MHP in Modes 1 and 2 and non-bending NP in Mode 3), lateral (C; contains non-bending NP in all Modes) and dorsolateral (D; contains DLHPs in Modes 2 and 3 and non-bending NP in Mode 1) regions. Elevated (elev) NP (B) has significantly more basal nuclei in the MHP-containing midline of Mode 1 than in the midline of Mode 3 that lacks a MHP (^*^; *p*=0.018). Elevated midline of Modes 2 and 3 show a similar but statistically non-significant trend (dashed line in B; *p*=0.078). Nuclear distribution does not differ significantly between Modes or between flat and elevated neural plate in other NP regions. Comparing NP regions with each other, the elevated midline of Mode 1 contains significantly more basal nuclei than lateral or dorsolateral regions (*p*=0.002), and the elevated midline of Mode 2 contains more basal nuclei than the lateral region (*p*<0.001). (**F-H**) Mean total NP width (i.e. distance along basal surface from midline to lateral edge, see E) is significantly greater when the NP is elevated (F; grey bars) than flat (F: green bars). NP sub-regions outside (G) and within (H) the neural fold show a similar increased width in elevated NP in most comparisons (^*^p<0.001). However, neurulation Modes differ only for NP within the elevated neural fold (H) where width increases significantly from Mode 1 to Mode 2, and from Mode 2 to Mode 3 (^**^p<0.001).

Nuclear position data were collected from three sections, spaced every 10 μm, within each of the flat and elevated neural plate levels. Since the diameter of neuroepithelial nuclei is approximately 8 μm, this spacing of the sections precluded repeat analysis of the same nucleus. Within each section, three specific areas were assessed: median hinge point (MHP), dorsolateral hinge points (DLHPs), and the straight lateral region (LAT), defined as indicated by the dotted lines in Fig. 2A and Fig. S1B-D. Five nuclei were scored per section in MHP and DLHP and eight per section in LAT. Sections were projected from a digital camera to a computer monitor, and the mid-point (apico-basally) of each nucleus was recorded as occurring in the apical, middle or basal thirds of the neuroepithelium (as shown in Fig. 2A). Proportional distribution of nuclei between the three apico-basal thirds formed the basis of the quantitative comparison of nuclear position.

Other morphometric comparisons were based on calibrated linear measurements and nuclear counts obtained from each half of three transverse sections, at flat and elevated PNP levels, in five embryos per Mode. Neuroepithelial width was measured along the basal surface, firstly along the neural fold, where adjacent to the surface ectoderm (nf in Fig. 1 K, L; Fig. 2E), and secondly along the remaining neural plate to the midline (npr in Fig. 1 K, L; Fig. 2E). Total neural plate width was the sum of nf and npr measurements. Data from replicate sections were pooled for each neural plate sub-division at each level of each embryo. Overall means+SEM were calculated for the 5 replicate embryos at each Mode. Cell numbers were determined by counting nuclei using the same neural plate sub-divisions as for width measurements. Nominal cell width was calculated by dividing mean neuroepithelial width by mean nuclear/cell number for the same region. Cell packing density was calculated by dividing the cell number of each neural plate region by its transverse sectional area, determined using the Measure/Analyse function of ImageJ.

### 2.3 Cell cycle analysis

In preliminary studies, bromodeoxyuridine was found to penetrate cultured embryos poorly, and so ^3^H-thymidine was used for the cell cycle analysis. Embryos were cultured for 1 h from E9.5 and then ^3^H-thymidine (25 Ci/mmol ) was added to a final concentration of 1 μCi/ml. For the main analysis, embryos were harvested from culture at 0.5 h time intervals up to a maximum of 8 h. Only embryos with 18-22 somites (Mode 2 neurulation) after culture were included in the analysis of cell cycle kinetics. Autoradiography was performed on 2.5 μm-thick plastic transverse sections through the PNP, using Ilford K5 emulsion diluted 1:2 with 2% glycerol solution. Nuclei were considered labelled if the grain count exceeded background level, determined from an adjacent non-cellular area of the same slide.

^3^H-thymidine labelling index (LI: number of labelled nuclei divided by total number of nuclei) was calculated from three sections per embryo and six embryos per time point. Analysis was performed on seven tissue regions as defined in Fig. 6A: MHP, LAT, DLHP, DL, notochord, dorsal surface ectoderm (DSE: surface ectoderm juxtaposed to neuroepithelium) and lateral surface ectoderm (LSE: an equal number of cells contiguous with the DSE, juxtaposed to paraxial mesoderm). A minimum of 900 cells was scored in the construction of each LI vs time curve for calculation of cell cycle parameters.

To determine the time between addition of ^3^H-thymidine to the culture, and first detection of incorporation in sections, embryos were harvested at 5-10 min intervals throughout the first hour of culture. Autoradiographic signal was readily visible after 30 min incubation (10/10 embryos) in a subset of cells in each of the tissues/regions examined. In contrast, 0/10 and 11/18 embryos harvested after 15 and 20 min exposure respectively exhibited labelling. Hence, labelling was considered to begin after 30 min exposure to ^3^H-thymidine in culture and the labelling period was set to t=0 at this time.

Median LI+SEM was plotted against time, and was found to rise until the entire cycling cell population was labelled (LI=1.0). Regression analysis was then performed on the LI values leading up to the plateau, generating a family of regression lines by including one additional time point after the plateau was first reached in each successive calculation (Fig. 6B). Of the regression lines thus generated for each data set, the line that most closely fitted the data (i.e. had the highest R^2^ value) was chosen for subsequent cell cycle analysis, using the method of Nowakowski et al. (1989). When a randomly proliferating cell population is exposed continuously to ^3^H-thymidine, the time taken for LI to reach a plateau is equivalent to the length of G2+M+G1. That is, the time taken for a cell that left S-phase immediately before the start of labelling to re-enter S-phase. Hence, to determine total cell cycle length (Tc), the period G2+M+G1 was added to the length of S-phase (Ts), which was calculated as y_0_/a, where a is the slope of the regression line and y_0_ is the intercept on the y-axis.

### 2.4 Vital cell labelling

*DiI-DiO double labelling.* E8.5 or E9.5 embryos were dissected for culture and positioned in a well cut in an agarose-bottomed petri dish. The caudal embryonic region was exposed through an opening in yolk sac and amnion. A glass micropipette controlled by a Leica micromanipulator was used to direct a gentle stream of DiO (Vybrant DiO; V-22886; Molecular Probes) onto the apical surface of the dorsal-most aspect of the neural plate, about mid-way along the PNP. Then a mark of DiI (CM-DiI; C-7001; Molecular Probes) was made on the apical surface of the neural plate at the same axial level, but more ventrally (~ 30% of distance from dorsal to ventral). Embryos with appropriately positioned, non-overlapping apical DiI and DiO marks were cultured for 5 h before extraembryonic membranes were removed. Analysis was by comparison of t=0 and t=5 side-view photographs taken on a Leica fluorescence stereo-microscope (Fig. S2).

#### 2.4.1 DiI single labeling

DiI was injected through the neural plate, about mid-way along the PNP and~60% of the distance from dorsal to ventral. A hand-held, mouth-controlled glass micropipette was inserted through the neural fold from the outside into the lumen of the PNP. A steady stream of DiI was expelled while withdrawing the pipette, to label neural plate and paraxial mesoderm cells along the track of the pipette. Embryos with a clear DiI track were randomly sampled immediately after labelling (t=0) or after 18 h culture (t=18). Following fixation in PFA, embryos were wax-embedded and transversely sectioned at 7 µm thickness. Sections were processed for anti-fibronectin immunofluorescence (rabbit polyclonal IgG; Abcam ab23750), stained with DAPI, mounted in Mowiol 4-88 mounting medium (Sigma-Aldrich, MO; prepared with glycerol and 0.2 M Tris pH 6.8), and examined and photographed by epifluorescence on an inverted LSM710 confocal system mounted on an Axio Observer Z1 microscope (Carl Zeiss Ltd, UK). Images were acquired as z-stacks using a 40× water immersion objective. Dorso-ventral distances along the neural plate in relation to the positions of DiI labelling were measured using the Measure/Analyse function of ImageJ.

### 2.5 Immunohistochemistry

Embryos were fixed for 24 h in cold 4% paraformaldehyde in PBS (PFA), washed x 3 in PBS, dehydrated, cleared in Histoclear (National Diagnostics), embedded in paraffin wax and sectioned at 8 μm thickness. Sections were dewaxed, microwaved in 0.1 M citric acid buffer, washed in PBS-Triton 0.3% (PBS-T) for 10 min, then treated with 3% hydrogen peroxide. Sections were blocked in PBS-T containing 5% FCS and 5% normal goat serum for 2 h. Primary antibodies were applied overnight at 4 °C: anti-Cdk4 (Sigma, C8218; 1:80 dilution), anti-Cyclin D1 (Sigma, C7464; 1:40 dilution) and anti-p27 (Sigma, P2092; 1:80 dilution). Secondary antibody was biotinylated rabbit anti-mouse IgG (1:400 dilution), detected using Avidin-Biotin-Complex/horseradish peroxidase (DAKO) and diaminobenzidine. Three embryos at each Mode of neurulation were assessed with each antibody.

### 2.6 Statistical analysis

Nuclear position data were analysed by chi-square test. Other quantitative data were compared using analysis of variance, Student’s t-test or non-parametric equivalents. When significant differences were found, data were subjected to pairwise post-hoc testing with protection of α-level. All statistical tests were performed using Sigmastat v3.2.

## 3 Results

Mouse neural tube closure is initiated at E8.5, at the hindbrain-cervical boundary (Closure 1), with neurulation propagating rostrally into the hindbrain, and caudally along the trunk, from this level. The posterior neuropore (PNP), thus formed, comprises a region of open neural folds caudal to the latest point of spinal neural tube closure (Fig. 1A,E,I). During axis development, the length of the PNP becomes reduced progressively (Fig. 1B,F,J) until it closes completely at the 30 somite stage (E10).

Apico-basal nuclear localisation, neural plate width, neural plate and surface ectodermal cell numbers, and nominal cell widths and density were analysed at two levels of each PNP: caudally, where the folding of the neural plate begins (‘flat’ neural plate; Fig. 1 C,G,K), and rostrally, where the neural folds are nearing completion of closure (‘elevated’ neural plate; Fig. 1D,H,L). The timing of the neurulation process at these two levels is separated by about 4-6 h of development. Three patterns of neural plate morphogenesis were analysed: Mode 1 (late E8.5)-only the MHP is formed; Mode 2 (E9.5)-both MHP and DLHPs are present; Mode 3 (E10)-only the DLHPs are present, and acute MHP bending is not present (Fig. 1).

### 3.1 Increased basal nuclear localisation in the MHP but not in DLHPs

Examination of plastic 2.5 µm-thick sections through the mouse PNP suggested that the MHP, but to a lesser extent the DLHP or non-bending neuroepithelium, is enriched for basally located nuclei (Fig. S1). To examine this question quantitatively, we scored numbers of nuclei in apical, middle or basal thirds of the neuroepithelium in transverse plastic sections (Fig. 2A). Whereas 30-40% of nuclei were basal in midline (MHP) cells of the flat neural plate at Modes 1 and 2, once the neural folds had elevated, around 60% of MHP nuclei were basal (Fig. 2B). Concomitantly, the MHP of the elevated neural plate showed a striking reduction in apically located nuclei (including all mitoses): the midline of the flat neural plate at Modes 1 and 2 had 20-30% apically located nuclei, whereas only 5-10% of nuclei were apical in the MHP of the elevated neural plate (Fig. 2B).

In contrast to Modes 1 and 2, embryos in Mode 3 neurulation, where the midline does not exhibit MHP bending, showed 40% basal nuclear localisation and 20% apical localisation in the midline at both flat and elevated levels (Fig. 2B). Similarly, non-bending lateral neuroepithelium (Fig. 1G,H) showed a uniform 30-40% basal and 20-30% apical nuclei at both flat and elevated levels, in all Modes of neurulation (Fig. 2C). Hence, there is no shift towards basal nuclear localisation in the absence of MHP bending.

In the dorsolateral region, the DLHPs within the elevated neural plate at Modes 2 and 3 contained 51% and 47% basal nuclei, respectively, compared with 37% in the non-bending dorsolateral region of Mode 1 (Fig. 2D). Proportions of apical nuclei in the dorsolateral region of elevated neural plate for Modes 1, 2 and 3 were 24%, 17% and 16% respectively. Hence, DLHP cells showed a trend towards enrichment of basal nuclei, but the proportions of nuclei in apical, middle and basal thirds of the dorsolateral neuroepithelium did not differ statistically between Modes 1, 2 and 3, nor between flat and elevated neural plate (Fig. 2D).

In conclusion, the MHP is associated with a significant increase in the proportion of basally located nuclei during neural fold elevation in mice. This is similar to findings in the chick embryo (Schoenwolf and Franks, 1984), and is consistent with a mechanism of midline bending based on a predominance of wedge shaped cells. There is a trend towards basal nuclear localisation also in the DLHP, but this is not as marked as in the MHP, and fails to reach statistical significance.

### 3.2 Neural plate width changes during neural fold elevation

We next assessed neural plate width and cell number in the transverse plane, to determine whether changes in these parameters correlate with DLHP formation. For this analysis, the neural plate was divided into two regions: (i) the neural fold, defined as the dorsolateral region of neural plate with basal surface juxtaposed to surface ectoderm (nf in Figs 1K, L; 2E); (ii) the ventromedial region outside the neural fold, with basal surface juxtaposed to paraxial mesoderm and notochord (npr in Figs 1K, L; 2E).

The width of the entire half-neural plate (Total NP) was greater in the elevated than flat neuroepithelium (Fig. 2F), and a similar change was evident in the neural plate sub-domains, outside and within the neural fold (Fig. 2G, H). However, neural plate width differed between the three Modes of neurulation only within the neural fold, whose width was markedly greater at Modes 2 and 3, when DLHPs are present, than at Mode 1 when DLHPs are absent (Fig. 2H). Thus, the width of the elevated neural fold constitutes 27%, 35% and 42% of total neural plate width in Modes 1, 2 and 3, respectively. In contrast, neuroepithelium outside the neural fold did not differ in width between Modes 1, 2 and 3 (Fig. 2G). Hence, the formation of elevated neural folds involves a widening of the neural plate, mainly within the neural fold that is in contact with surface ectoderm.

### 3.3 Cell number changes during neural fold elevation

**Fig. 3:**
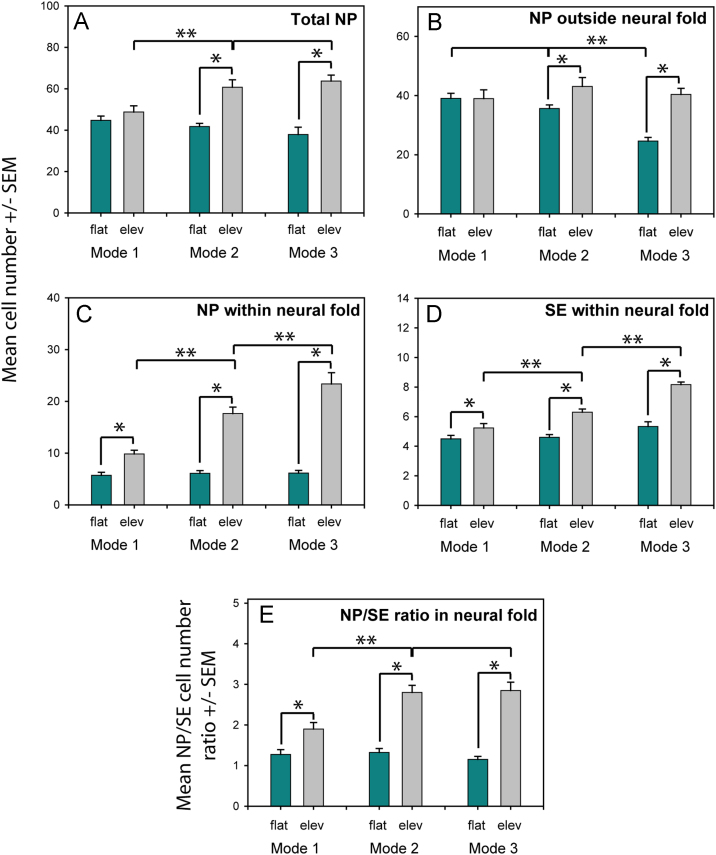
Cell number in neural plate (NP) and juxtaposed surface ectoderm (SE) during neural plate bending. (**A-D**) Mean cell number in the entire half-NP (A), in NP sub-domains outside (B) and within (C) the neural fold, and in SE juxtaposed to the NP (D). Elevated NP and SE (grey bars) contain significantly more cells than flat NP and SE (green bars) in most comparisons (^*^p<0.001). Cell number of elevated NP in the neural fold (C) and juxtaposed SE (D) increase significantly from Mode 1 to Mode 2, and from Mode 2 to Mode 3 (^**^p<0.001). Cell number in total elevated NP (A) also increases significantly from Mode 1 to Modes 2 and 3 (^**^p=0.014*).* In contrast, cell number in NP outside the neural fold (B) varies with neurulation mode only for flat NP (^**^p=0.006). (**E**) NP/SE cell number ratio within neural fold increases significantly from flat to elevated neural plate (^*^p<0.001). Within the elevated neural fold, NP/SE ratio is significantly greater in Modes 2 and 3, which have DLHPs, than in Mode 1 which lacks DLHPs (^**^p=0.007).

Neural plate cell (nuclear) number increased from flat to elevated neuroepithelium mainly in Modes 2 and 3 of neurulation (Fig. 3A-C). Moreover, this cell number increase was most dramatic within the neural fold, where the transition from flat to elevated neural plate was marked by a 5-fold increase in cell number at Mode 3, compared with only a 2-fold increase at Mode 1 (Fig. 3C). In contrast, much smaller cell number increases occurred during elevation outside the neural fold, and cell number did not differ significantly in this region between the elevated neural plate of Modes 1, 2 and 3 (Fig. 3B).

A significantly greater number of surface ectoderm cells (red arrowheads in Fig. 1K) were juxtaposed to the elevated than flat neural fold, with a progressively greater cell number increase from Modes 1 to 3 (Fig. 3D). However, this cell number increase was not as dramatic as in adjacent neuroepithelium, so the ratio of neural plate to surface ectoderm cell number was significantly greater in Modes 2 and 3 than in Mode 1 (Fig. 3E). Hence, as the DLHP forms there is a 2 to 3-fold increase in the number of neural plate cells adjacent to each surface ectoderm cell in the dorsolateral neuroepithelium.

### 3.4 Decreased cell width and increased cell density in the elevating neural fold

**Fig. 4:**
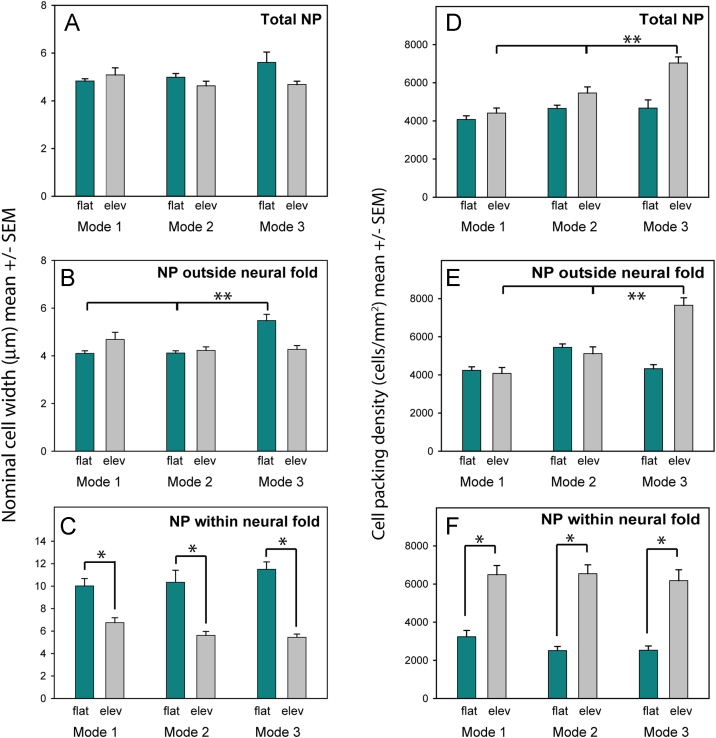
Nominal cell width and cell packing density in the neural plate (NP) during bending. (**A-C**) Mean nominal cell width within the neural fold (C) diminishes by 50% from flat (green bars) to elevated (grey bars) NP for all Modes of neurulation (^*^ in C; *p*<0.001), whereas there is no comparable cell width change with neural fold elevation in total NP (A) or in NP outside the neural fold (B). Nominal cell width outside the neural fold is statistically greater in flat NP of Mode 3 than in Modes 1 or 2 (^**^ in B; *p*<0.001). (**D-F**) Mean cell density within the neural fold (F) increases significantly from flat to elevated neural plate at each Mode of neurulation (^*^p<0.001) but does not change significantly in total NP (D) or in NP outside the neural fold (E). Cell density in total NP and in NP outside the neural fold is statistically greater in elevated NP of Mode 3 than in Modes 1 or 2 (^**^ in D, E; *p*=0.001).

Because the neuroepithelium is pseudostratified, with nuclei at different apico-basal levels (Fig. S1), we divided neuroepithelial width by nuclear number to yield a nominal ‘cell width’ value. We also independently calculated cell packing density by dividing the neuroepithelial area, measured from transverse sections, by the cell number within it. Nominal cell width did not change significantly between flat and elevated neural plate in the entire half-neural plate (Fig. 4A) or outside the neural fold (Fig. 4B), whereas the neuroepithelium within the neural fold showed an approximate 2-fold reduction in nominal cell width between flat and elevated neural plate (Fig. 4C). Cell packing density showed the opposite trend: the neuroepithelium within the neural fold showed a 2 to 3-fold increase in cell density as the neural folds elevated (Fig. 4F), a change which was not seen in the entire neural plate or neuroepithelium outside the neural fold (Fig. 4D, E). Hence, cells become increasingly closely packed in the dorsolateral neural plate as the folds elevate and DLHPs form.

### 3.5 Dorso-ventral variation in cell proliferation

We asked whether differences in proliferation rate might be responsible for the cellular changes we observed during neural fold elevation. Cell cycle-related marker expression was evaluated by immunohistochemistry, and E9.5 mouse embryos were labelled in culture with ^3^H-thymidine to enable cell cycle length calculation

**Fig. 5:**
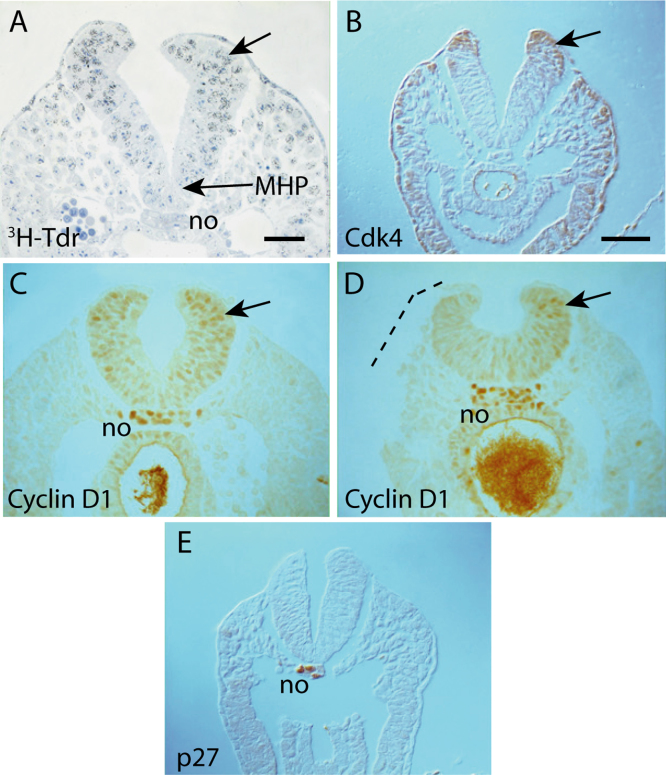
Markers of cell cycle variation during neural plate bending. (**A**) ^3^H-thymidine incor*p*oration in the PNP at Mode 2, following 2 h exposure *in vitro*. Incorporation intensity (black autoradiographic grains on Toluidine Blue stained background) appears highest dorsolaterally (arrow) and lowest at the MHP. (**B-E**) Immunohistochemistry for Cdk4 (B), Cyclin D1 (C, D) and p27 (E). The dorsolateral neural plate exhibits the most intense signal for both Cdk4 and Cyclin D1 (arrows in B-D), consistent with enhanced ^3^H-thymidine incorporation dorsally. When the surface ectoderm (DSE and LSE: see Fig. 5A) is removed surgically from the PNP region (dashed line in D), Cyclin D1 expression is lost within 30 min. The cell cycle inhibitor p27 (E) is expressed solely in the notochord (no) consistent with its prolonged cell cycle time. Serial sections from a minimum of three embryos were analysed with each antibody. Scale bars: 25 μm in A; 50  μm in B (also C-E).

A dorso-ventral difference in ^3^H-thymidine incorporation was evident in the PNP of a Mode 2 embryo following 2 h labelling *in vitro* (Fig. 5A). Moreover, Cdk4 expression was detected in the dorsal neural plate and adjacent surface ectoderm (Fig. 5B). Cyclin D1, which interacts with Cdk4 during G1 to S transition of the cell cycle (Sherr and Roberts, 2004), was widely expressed in the neuroepithelium, with an apparent dorsal-to-ventral gradient of expression intensity (Fig. 5C). The notochord also showed intense Cyclin D1 expression (Fig. 5C) but this was co-expressed with the cell cycle inhibitor p27 (Fig. 5E), perhaps accounting for the notochord’s diminished cell proliferative activity (Smith and Schoenwolf, 1987; Copp et al., 1988).

**Fig. 6:**
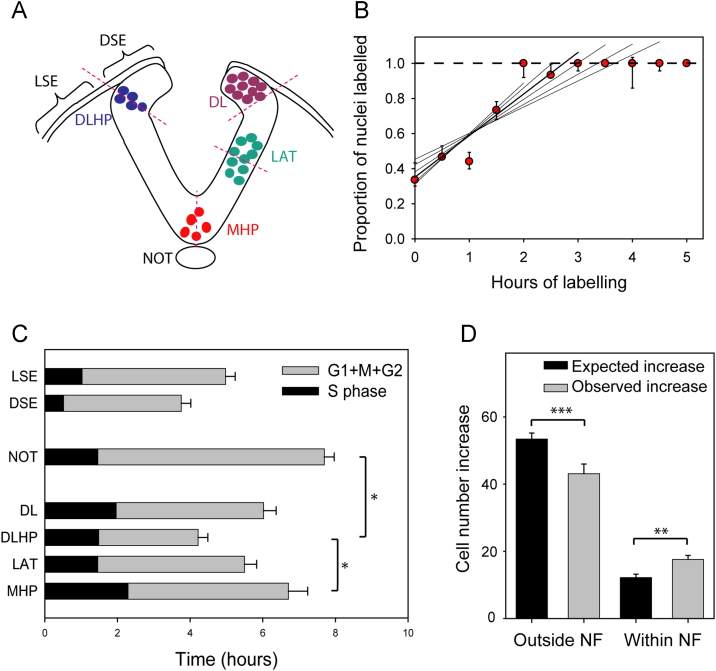
Cell cycle analysis during neural plate bending. (**A**) Diagram of elevated Mode 2 neural plate with attached surface ectoderm, showing tissues included in the analysis: DL, dorsolateral neural plate; DLHP, dorsolateral hinge point; DSE, dorsal surface ectoderm; LAT, lateral non-bending neural plate; LSE, lateral surface ectoderm; MHP, median hinge point; NOT, notochord. (**B**) Representative data from a ^3^H-thymidine labelling experiment. An increasing proportion of nuclei are labelled by continuous exposure to ^3^H-thymidine until, in this example, 100% of nuclei are labelled after 2 h. Regression analysis yields a family of lines, each generated by including one additional post-2 h time point. The best-fit regression line (bold) was chosen to calculate cell cycle length in each data set. See also Fig. S3. (**C**) Total cell cycle length (hours+SEM) as calculated for tissues in (A). Calculated lengths of S phase (black) and combined G1+M+G2 (grey) are shown. Total cell cycle length varies significantly between tissues (*p*<0.001; 1-way ANOVA), with DLHP exhibiting a significantly shorter cell cycle length than either MHP or notochord (^*^p<0.05; post-hoc Bonferroni t-tests). (**D**) Cell number increase in neural plate between flat and elevated stages of PNP closure (data from Table 1). Expected increase (black bars) is based on cell cycle length and observed increase (grey bars) is based on cell number counts. Expected increase significantly exceeds observed for neural plate outside the neural fold (NF)(^***^p=0.02; t-test), whereas expected increase is significantly less than observed for neural plate within the NF (^**^p=0.01).

Continuous exposure of cultured E9.5 embryos to ^3^H-thymidine resulted in 100% of cells becoming labelled in all embryonic tissues studied (Fig. 6A, B). Cell cycle analysis (Nowakowski et al., 1989) revealed significant variation in cell cycle length between tissues of the PNP (*p*<0.001) (Fig. S3). Dorsal surface ectoderm (DSE) and DLHP had the shortest cell cycles: 3.8 and 4.2 h respectively, whereas the MHP and notochord had the longest cell cycles: 6.7 and 7.7 h respectively (Fig. 6C). In the MHP, the length of S phase, calculated at 2.3 h, was the longest of all the tissues studied in the PNP. This is consistent with the small proportion of apically located nuclei (including all mitoses) in the MHP (Fig. 2B).

### 3.6 Cyclin D1 gradient is maintained by interaction with the surface ectoderm

Surgical removal of the surface ectoderm from the neural fold abolishes DLHP formation within 5 h (Ybot-Gonzalez et al., 2002). Embryos whose surface ectoderm had been removed showed a decrease in Cyclin D1 immunoreactivity within 30 min of operation (Fig. 5D). Hence, embryos lacking surface ectoderm on the outside of the neural fold no longer exhibit a dorso-ventral gradient of Cyclin D1 expression. We conclude that the increased proliferation of dorsal neuroepithelium in the closing neural tube likely depends on an interaction with the attached surface ectoderm.

### 3.7 Cell number increase in neural folds cannot be explained solely by cell proliferation

The marked neuroepithelial cell number increase during elevation of the DLHP-containing neural fold at Modes 2 and 3 (Fig. 3C) correlated with the finding of a particularly short cell cycle length in this neural plate region (Fig. 6C). The transition from flat to elevated neural plate in the present study encompassed 4-6 h of development, sufficient for the completion of one cell cycle in the dorsolateral neural plate and less than one cell cycle in the ventromedial neural plate, based on the calculated cell cycle times. In Mode 2, the neural fold contained an average of 6.1 neuroepithelial cells per transverse section at the flat neural plate stage. One cell doubling should generate 12.2 cells, and yet the elevated neural fold contained an average of 17.6 neuroepithelial cells (Table 1). In contrast, the neuroepithelium outside the neural fold increased from 35.6 to 43.1 cells between flat and elevated neural plate stages, whereas even with only a 0.5 cell doubling (a likely under-estimate), this sub-population should have generated 53.4 cells. Hence, there is a greater than expected increase in cell number in the neural fold, and a less than expected increase outside the neural fold, based on the cell cycle data (Table 1; Fig. 6D). A possible explanation for this discrepancy is that neuroepithelial cells translocate ventro-dorsally between neural plate domains as the neural folds elevate.

### 3.8 Vital cell labelling reveals ventral-to-dorsal cell translocation as the neural folds elevate

Vital cell labelling was performed to test experimentally whether neuroepithelial cells translocate into the dorsal neural folds as neurulation progresses. In a preliminary experiment (Fig. S2), the apical surface of one neural fold received two focal marks: a dorsal DiO mark near the neural fold tip and a more ventral DiI mark, about 30% below the neural fold tip. Embryos at E8.5 (Mode 1; no DLHPs) showed no increase in overlap of the DiO and DiI marks after 5 h culture, whereas the majority of embryos at E9.5 (Mode 2; DLHPs present) showed dorsal extension of the DiI mark to overlap with the dorsal DiO mark after 5 h culture (Fig. S2). While this result supported a possible ventral-to-dorsal translocation of neuroepithelial cells during DLHP formation, the experiment involved only short-term culture, owing to the need to open the extraembryonic membranes, and was not analysed in embryo sections, due to the instability of DiO during histological processing.

**Fig. 7:**
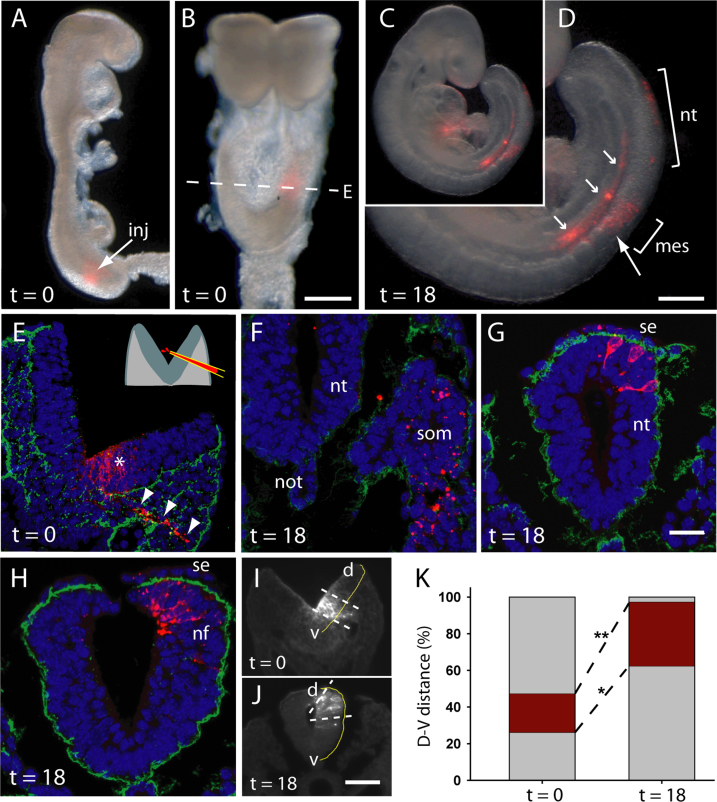
Long-term vital cell labelling of the neuroepithelium. (**A, B**) Late E8.5 embryos at t=0. Lateral (A) and dorsal (B) views show site of focal DiI (red) labelling. Arrow (inj): site at which micropipette was inserted to label the full thickness of the neural fold. (**C, D**) Labelled embryo at t=18 showing typical distribution of DiI-labelled cells in mesoderm (mes) and neural tube (nt) along the rostro-caudal axis. Large arrow: original level of labelling. Small arrows: intense ectopic labelling along dorsal aorta. (**E**) Transverse section (DiI: red; fibronectin: green; nuclei: DAPI) of embryo at t=0, along dashed line in B, showing track of labelled cells through neuroepithelium (asterisk) and paraxial mesoderm (arrowheads). Inset: trans-neural fold injection method. (**F-H**) Transverse sections of embryos at t=18, showing DiI labelling in somitic (som) mesoderm (F), and dorsal neuroepithelial cells within closed (G) and open (H) neural tube. Many labelled neural tube cells are adjacent to surface ectoderm (se). (**I-K**) Quantitation of dorso-ventral extent of DiI labelling in neural plate/tube. Sections (I, J) show ventral (v) and dorsal (d) borders of DiI (dashed lines) in relation to total ventral-to-dorsal distance (yellow lines). Graph (K) shows significant dorsal translocation of cells at t=18 compared with t=0 for both ventral (^*^p=0.005) and dorsal (^**^p<0.001) limits of DiI labelling (n=9 embryos for each time point). Mean values (+ SEM): t=0, v=26.2+7.4, d=47.4+7.5; t=18, v=62.4+3.9, d=97.4+1.6. Abbreviation: not, notochord. Total number of embryos analysed: 19 at t=0; 20 at t=18. Scale bars: A, B: 0.25 mm; D: 0.5 mm; E-H: 0.02 mm; I, J: 0.04 mm.

We therefore performed a second experiment in which DiI labelling was achieved by trans-neural fold injection, without opening the extraembryonic membranes, enabling longer-term culture and subsequent histological analysis (Fig. 7A-D). Both neuroepithelium and adjacent paraxial mesoderm (Fig. 7E) contained DiI-labelled cells in 14/19 injected embryos that were fixed within 1 h of injection (t=0). In embryos cultured for 18 h (t=18), DiI-labelled mesoderm cells were found within the somite and/or lateral plate mesoderm near the site of injection (Fig. 7F). In contrast, DiI-labelled neuroepithelial cells were located more caudally, along the recently closed neural tube and/or neural folds of the open PNP (in 10/20 injected embryos; Fig. 7D, G, H). The DiI-labelled neuroepithelial cells occupied a significantly more dorsal position in the neural tube/fold at t=18 than at t=0 (compare Figs. 7E and 7 G, H), with a statistically significant difference after quantitation (Fig. 7I-K). These results are consistent with the ventro-dorsal translocation of cells in the DLHP-containing neuroepithelium as the neural folds elevate prior to closure.

## 4 Discussion

We have examined the cellular events that accompany dorsolateral bending of the mouse neural plate, by which the neural folds are brought into apposition in the dorsal midline. Whereas bending at the MHP is characterised by neuroepithelial cells with basal nuclear localisation and a relatively long cell cycle, the DLHP exhibits neither of these properties. DLHP cells are the most rapidly proliferating in the neuroepithelium, and show an apico-basal nuclear distribution that does not differ significantly from non-bending neural plate. This suggests that neuroepithelial bending occurs by different cellular mechanisms at the MHP and DLHPs.

### 4.1 Differences in the mechanisms of midline and dorsolateral bending

The MHP develops in midline neuroepithelium immediately overlying the notochord, and MHP induction requires proximity (or perhaps physical attachment) to the notochord, with likely inducing factors including Shh and Chordin (Van Straaten et al., 1985; Smith and Schoenwolf, 1989; Placzek et al., 1990; Davidson et al., 1999; Ybot-Gonzalez et al., 2002; Patten and Placzek, 2002). By contrast, DLHP formation persists in the absence of both ventral Shh and dorsal BMP signalling (Ybot-Gonzalez et al., 2007), and can continue even in embryos from which the paraxial mesoderm has been surgically removed (Ybot-Gonzalez et al., 2002).

The factor that appears critical for DLHP formation is a physical association between the neuroepithelium and surface ectoderm. In the present study, we found that enhanced cell proliferation, which characterises the dorsolateral neural plate, depends on neuroepithelial contact with the surface ectoderm. Surgical removal of the surface ectoderm leads to loss of DLHPs, and prevents new dorsolateral bending, whereas retention of only a small fragment of surface ectoderm is sufficient for bending to occur (Ybot-Gonzalez et al., 2002). Similar findings were reported for chick and amphibian embryos (Jacobson and Moury, 1995; Moury and Schoenwolf, 1995), suggesting a conserved morphogenetic mechanism among vertebrates. Moreover, the finding that relatively few surface ectoderm cells need to be attached to the neuroepithelium for DLHP formation to occur, argues against a mechanism of DLHP formation involving a ‘push force’ from the expanding surface ectoderm (Alvarez and Schoenwolf, 1992). It seems more likely that the surface ectoderm exerts a localised signalling or contact-mediated influence on the adjacent neuroepithelium to enable DLHP formation. It remains to be determined what are the key surface ectodermal factors or properties that induce enhanced cell proliferation, and encourage ventral-to-dorsal cell translocation into the dorsolateral neural plate.

### 4.2 Neuroepithelial cells translocate dorsolaterally during neural fold elevation

Medio-lateral neuroepithelial width and cell number both increase during neural fold elevation, but these changes are not uniform: they are observed mainly in neuroepithelium that contacts the surface ectoderm, not in more ventro-medial neural plate adjacent to paraxial mesoderm and notochord. While neuroepithelial cell proliferation is most rapid dorsolaterally, the cell number increase in this region cannot be accounted for solely by local cell proliferation. Moreover, mitoses are mainly oriented in the rostro-caudal plane of the mouse and chick neuroepithelium (Sausedo et al., 1997), suggesting that cell proliferation alone is unlikely to contribute significantly to medio-lateral expansion of the neural plate.

These findings led us to hypothesise that some neuroepithelial cells may translocate in a ventral-to-dorsal direction to become associated with the surface ectoderm by the elevated neural plate stage. Support for this idea comes from finding a 3-fold increase in neural plate to surface ectoderm cell number ratio, and a 2-fold increase in cell packing density, as DLHPs form. Moreover, our vital cell labelling experiments provide strong experimental evidence in favour of this morphogenetic cell translocation. Ventral neuroepithelial cells labelled with DiI early in the closure process adopt a more dorsal position as neural tube closure progresses to completion.

### 4.3 Buckling: a possible mechanism of neural plate morphogenesis

**Fig. 8:**
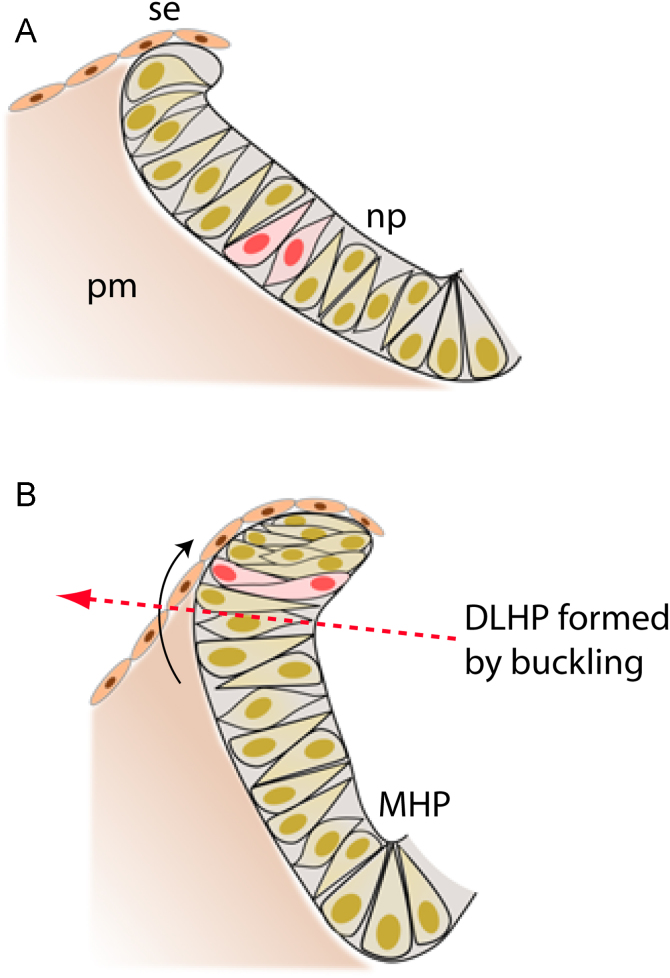
A model of DLHP bending during neural fold elevation. Diagrammatic transverse sections of flat (**A**) and elevated (**B**) half-neural plate (np), juxtaposed surface ectoderm (se) and paraxial mesoderm (pm). As the neural fold elevates, neuroepithelial cells translocate ventro-dorsally (curved arrow in B) so that increasing numbers contact the surface ectoderm in the elevated neural fold. This results in increased cell density and diminished cell width within the dorsal neural fold region. Two cells originating ventrally (pink) are depicted translocating dorsally, as was observed in the DiI vital cell labelling study. Cells at the midline (MHP) exhibit basal nuclear localisation, whereas elsewhere in the neuroepithelium nuclei are observed at all apico-basal positions. DLHP formation is suggested to result from physical ‘buckling’ of the neural plate (red arrow) at the junction between the dorsal region of increased cell density and ventral region of lower cell density.

It is striking that DLHPs form precisely at the dorso-ventral level where the neural plate transitions from basal contact with paraxial mesoderm to basal contact with surface ectoderm. We found that, during DLHP formation, both cell number and cell density increase markedly, while nominal cell width decreases, particularly in the dorsal neural fold component of the neuroepithelium. This might suggest a mechanism of neural plate bending (Fig. 8) in which the elevating neuroepithelium behaves biomechanically as a biphasic structure. That is, each half-neural plate comprises a dorsolateral component of increasing cell density in direct physical contiguity with a ventromedial component of relatively constant, lower cell density. We suggest, therefore, that DLHP formation could represent the inward (i.e. medially directed) ‘buckling’ of the neuroepithelium at the phase transition point (see red arrow: ‘basal contact transition point’ in Fig. 8). This represents a markedly different view of neuroepithelial morphogenesis from the rather uniform bending that is usually envisaged to result from generalised apical constriction of the neuroepithelium (Sawyer et al., 2010).

Buckling in architectural and skeletal structures is generally an unwanted effect of high compression forces that exceed the structure’s mechanical capacity. In contrast, tissue buckling during morphogenesis can be an integral part of organ development. In the embryonic intestine, villi form through the luminal buckling of the growing tubular epithelium as a result of mechanical constraints imposed by outer tissues, particularly the smooth muscle layers (Shyer et al., 2013) and the gut mesenchyme (Ben Amar and Jia, 2013). Furthermore, the overall buckling of growing tubes has been found to result from mechanical constraints imposed by adjacent, attached tissues in such diverse systems as the intestine, epididymis and plant roots, with the latter two structures forming helical loops as a result of buckling (Savin et al., 2011; Silverberg et al., 2012; Hirashima, 2014).

Perhaps most relevant to neural plate bending are previous studies that suggest epithelial buckling may result from regional heterogeneity in tissue proliferative activity. For example, in the chick, formation of the tubular embryonic gut from its ventrally-open precursor is associated with marked regional differences in cell proliferation rate that correlate with the pattern of endodermal folding. Lateral domains of high cell proliferation flank a midline domain with significantly lower proliferation. The proliferative differential was seen as forcing buckling of endoderm into lateral folds which eventually join and enclose a tube (Miller et al., 1999). In our study, an analogous difference in proliferative rate was also observed between dorsal and ventral neuroepithelium, but we conclude that proliferation differences alone are probably not sufficient to generate localised buckling. Rather, this occurs at sites of mechanical discontinuity within the neuroepithelium, caused by net cell translocation dorsally into the neural fold.

## 5 Conclusions

We have confirmed that, as in the chick, the midline of the mouse neural plate exhibits localised basal nuclear localisation, supporting a mechanism of MHP bending through cell wedging. However, the paired DLHPs form by a different cellular mechanism from the MHP, consistent with the known differences in the molecular regulation of these two bending events. Cell translocation occurs in a ventral-to-dorsal direction within the plane of the neuroepithelium, as the neural folds elevate. Together with an enhanced cell proliferative rate within the dorsal neuroepithelium, this results in disproportionate enlargement of the dorsal sub-region of the neural plate, where cells contact the surface ectoderm. Cells appear constrained within this region, as their nominal width decreases and their packing density increases as neurulation progresses. These findings suggest a mechanism of DLHP bending morphogenesis based on local buckling of the neuroepithelium at the transition point between the two neural plate sub-regions.

## Figures and Tables

**Table 1:** Cell numbers within and outside the neural fold during the transition from flat to elevated neural plate in Mode 2 neurulation: comparison of observed mean values with those expected from measured cell cycle lengths.

	**Flat neural plate**	**Elevated neural plate**			
	OBS ⁎	OBS ⁎	EXP ⁎⁎	OBS-EXP (mean)	*p* value ⁎⁎⁎
Within neural fold	6.1+0.5	17.6 ⁎⁎⁎+1.2	12.2 ⁎⁎⁎+1.0	+5.4	0.01
Outside neural fold	35.6+1.2	43.1+ 2.9	53.4 ⁎⁎+ 1.8	-10.3	0.02

⁎⁎ Observed (OBS) cell numbers (mean+SEM) are derived from 3 sections through both flat and elevated levels of neural plate from each of 5 embryos.

⁎⁎ Expected (EXP) cell numbers (mean+SEM) assume 1.0 cell doublings within the neural fold and 0.5 cell doublings outside the neural fold, from flat to elevated neural plate, as found in the cell cycle analysis (see Fig. 6).

⁎⁎⁎ Statistical analysis: OBS and EXP mean cell numbers in elevated neural plate were compared by Student’s t-test. Within the neural fold, significantly more cells are observed than expected based on measured cell doubling time (*p*=0.01). Conversely, outside the neural fold, significantly fewer cells are observed than expected based on doubling time (*p*=0.02).

## References

[bib1] Alvarez I.S., Schoenwolf G.C. (1992). Expansion of surface epithelium provides the major extrinsic force for bending of the neural plate. J. Exp. Zool.

[bib2] Ben Amar M., Jia F. (2013). Anisotropic growth shapes intestinal tissues during embryogenesis. Proc. Natl. Acad. Sci. U. S. A.

[bib3] Cockroft D.L., Copp A.J., Cockroft D.L. (1990). Dissection and culture of postimplantation embryos. Postimplantation Mammalian Embryos.

[bib4] Copp A.J. (2005). Neurulation in the cranial region-normal and abnormal. J. Anat..

[bib5] Copp A.J., Brook F.A., Roberts H.J. (1988). A cell-type-specific abnormality of cell proliferation in mutant (curly tail) mouse embryos developing spinal neural tube defects. Development.

[bib6] Davidson B.P., Kinder S.J., Steiner K., Schoenwolf G.C., Tam P.P.L. (1999). Impact of node ablation on the morphogenesis of the body axis and the lateral asymmetry of the mouse embryo during early organogenesis. Dev. Biol..

[bib7] Elms P., Siggers P., Napper D., Greenfield A., Arkell R. (2003). Zic2 is required for neural crest formation and hindbrain patterning during mouse development. Dev. Biol.

[bib8] Hirashima T. (2014). Pattern formation of an epithelial tubule by mechanical instability during epididymal development. Cell Rep..

[bib9] Jacobson A.G., Moury J.D. (1995). Tissue boundaries and cell behavior during neurulation. Dev. Biol.

[bib10] Langman J., Guerrant R.L., Freeman B.G. (1967). Behaviour of neuro-epithelial cells during closure of the neural tube. J. Comp. Neurol.

[bib11] Miller S.A., Adornato M., Briglin A., Cavanaugh M., Christian T., Jewett K., Michaelson C., Monoson T., Price F., Tignor J., Tyrell D. (1999). Domains of differential cell proliferation suggest hinged folding in avian gut endoderm. Dev. Dyn.

[bib12] Moury J.D., Schoenwolf G.C. (1995). Cooperative model of epithelial shaping and bending during avian neurulation: Autonomous movements of the neural plate, autonomous movements of the epidermis, and interactions in the neural plate epidermis transition zone. Dev. Dyn.

[bib13] Nowakowski R.S., Lewin S.B., Miller M.W. (1989). Bromodeoxyuridine immunohistochemical determination of the lengths of the cell cycle and the DNA-synthetic phase for an anatomically defined population. J Neurocytol..

[bib14] Patten I., Placzek M. (2002). Opponent activities of Shh and BMP signaling during floor plate induction in vivo. Curr. Biol..

[bib15] Placzek M., Tessier-Lavigne M., Yamada T., Jessell T., Dodd J. (1990). Mesodermal control of neural cell identity: Floor plate induction by the notochord. Science.

[bib16] Sauer F.C. (1935). Mitosis in the neural tube. J. Comp. Neurol.

[bib17] Sausedo R.A., Smith J.L., Schoenwolf G.C. (1997). Role of nonrandomly oriented cell division in shaping and bending of the neural plate. J. Comp. Neurol.

[bib18] Savin T., Kurpios N.A., Shyer A.E., Florescu P., Liang H., Mahadevan L., Tabin C.J. (2011). On the growth and form of the gut. Nature.

[bib19] Sawyer J.M., Harrell J.R., Shemer G., Sullivan-Brown J., Roh-Johnson M., Goldstein B. (2010). Apical constriction: A cell shape change that can drive morphogenesis. Dev. Biol.

[bib20] Schoenwolf G.C., Franks M.V. (1984). Quantitative analyses of changes in cell shapes during bending of the avian neural plate. Dev. Biol.

[bib21] Sherr C.J., Roberts J.M. (2004). Living with or without cyclins and cyclin-dependent kinases. Genes Dev..

[bib22] Shum A.S.W., Copp A.J. (1996). Regional differences in morphogenesis of the neuroepithelium suggest multiple mechanisms of spinal neurulation in the mouse. Anat. Embryol.

[bib23] Shyer A.E., Tallinen T., Nerurkar N.L., Wei Z., Gil E.S., Kaplan D.L., Tabin C.J., Mahadevan L. (2013). Villification: how the gut gets its villi. Science.

[bib24] Silverberg J.L., Noar R.D., Packer M.S., Harrison M.J., Henley C.L., Cohen I., Gerbode S.J. (2012). 3D imaging and mechanical modeling of helical buckling in Medicago truncatula plant roots. Proc. Natl. Acad. Sci. U. S. A.

[bib25] Smith J.L., Schoenwolf G.C. (1987). Cell cycle and neuroepithelial cell shape during bending of the chick neural plate. Anat. Rec.

[bib26] Smith J.L., Schoenwolf G.C. (1988). Role of cell-cycle in regulating neuroepithelial cell shape during bending of the chick neural plate. Cell Tissue Res..

[bib27] Smith J.L., Schoenwolf G.C. (1989). Notochordal induction of cell wedging in the chick neural plate and its role in neural tube formation. J. Exp. Zool.

[bib28] Smith J.L., Schoenwolf G.C., Quan J. (1994). Quantitative analyses of neuroepithelial cell shapes during bending of the mouse neural plate. J. Comp. Neurol.

[bib29] Van Straaten H.W.M., Thors F., Wiertz-Hoessels J., Hekking J., Drukker J. (1985). Effect of a notochordal implant on the early morphogenesis of the neural tube and neuroblasts: Histometrical and histological results. Dev. Biol..

[bib30] Ybot-Gonzalez P., Cogram P., Gerrelli D., Copp A.J. (2002). Sonic hedgehog and the molecular regulation of neural tube closure. Development.

[bib31] Ybot-Gonzalez P., Copp A.J. (1999). Bending of the neural plate during mouse spinal neurulation is independent of actin microfilaments. Dev. Dyn..

[bib32] Ybot-Gonzalez P., Gaston-Massuet C., Girdler G., Klingensmith J., Arkell R., Greene N.D., Copp A.J. (2007). Neural plate morphogenesis during mouse neurulation is regulated by antagonism of BMP signalling. Development.

