# Supplementary material for: Cellular basis of neuroepithelial bending during mouse spinal neural tube closure
Source: Dev Biol. 2015 Aug 15;404(2):113–24. doi: 10.1016/j.ydbio.2015.06.003 (PMC4528075; doi:10.1016/j.ydbio.2015.06.003)
Supplement: Supplementary file 1 — Supplementary material [file mmc1.docx]

SUPPLEMENTARY FIGURES

**
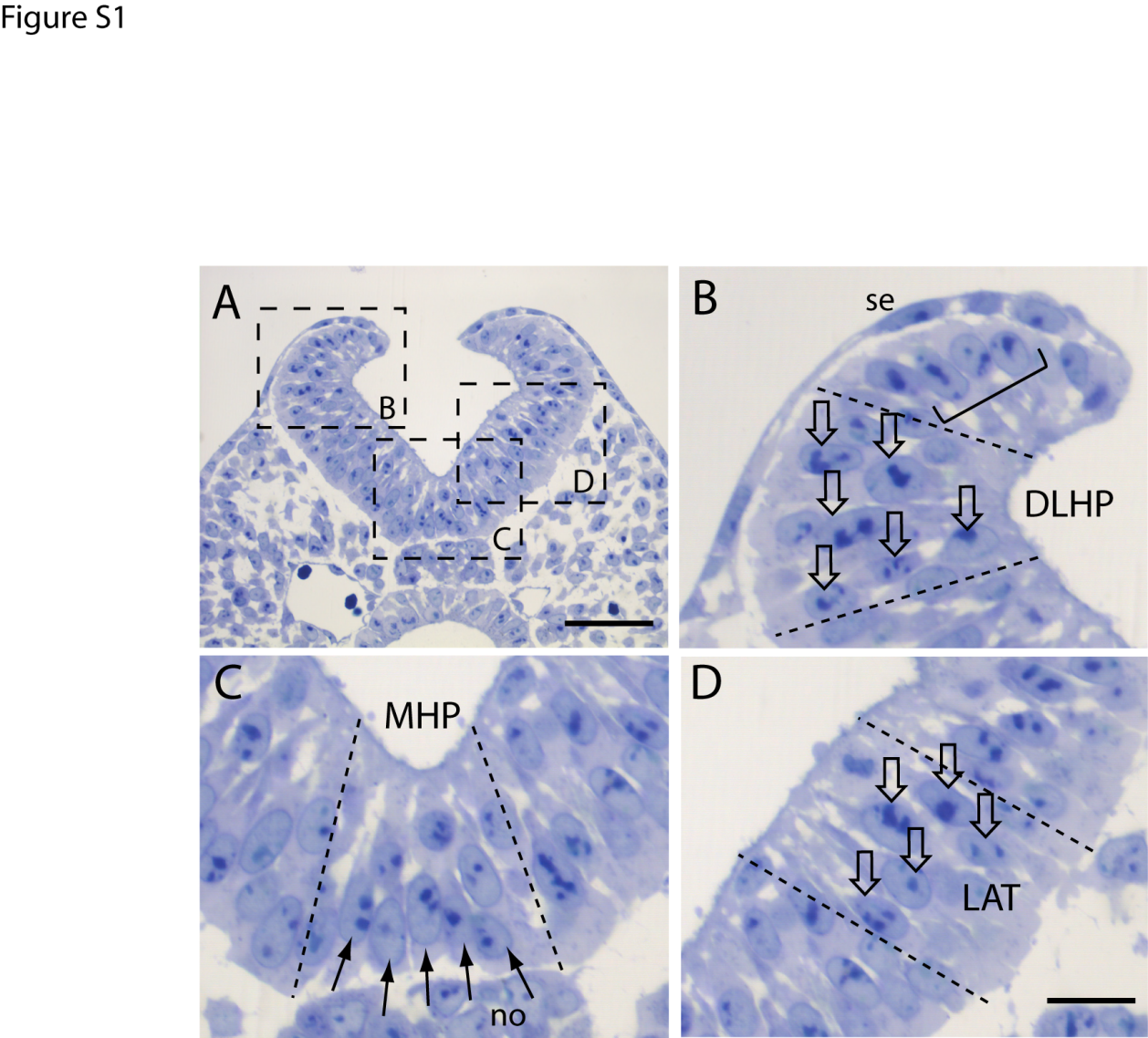
**

**Figure S1. Nuclear position differs between MHP and other neural plate regions.**Transverse 2.5 µm-thick plastic section (toluidine blue stain) through the elevated neural folds of a Mode 2 PNP. Dotted lines in B-D show the demarcation of the DLHPs, MHP and LAT regions as used in the statistical analysis (see Figure 2A-D). (**A**) Low magnification image showing the MHP and bilateral DLHPs. Enlarged regions in B-D are indicated by dashed boxes. (**B**) DLHP region showing nuclei at all apico-basal levels at the site of bending (open arrows). In this section, nuclei outside the DLHP dorsally appear more basal and close-packed (bracket), although the apical ends of these cells are not in the area of DLHP bending. Note the close association of the dorsolateral neural plate with juxtaposed surface ectoderm (se). (**C**) MHP region showing predominantly basal nuclei (solid arrows) in midline cells immediately overlying the notochord (no). (**D**) Lateral (LAT), non-bending neural plate showing nuclei at all apico-basal levels (open arrows). Scale bars: 0.6 mm (A); 0.2 mm (D, also for B, C).


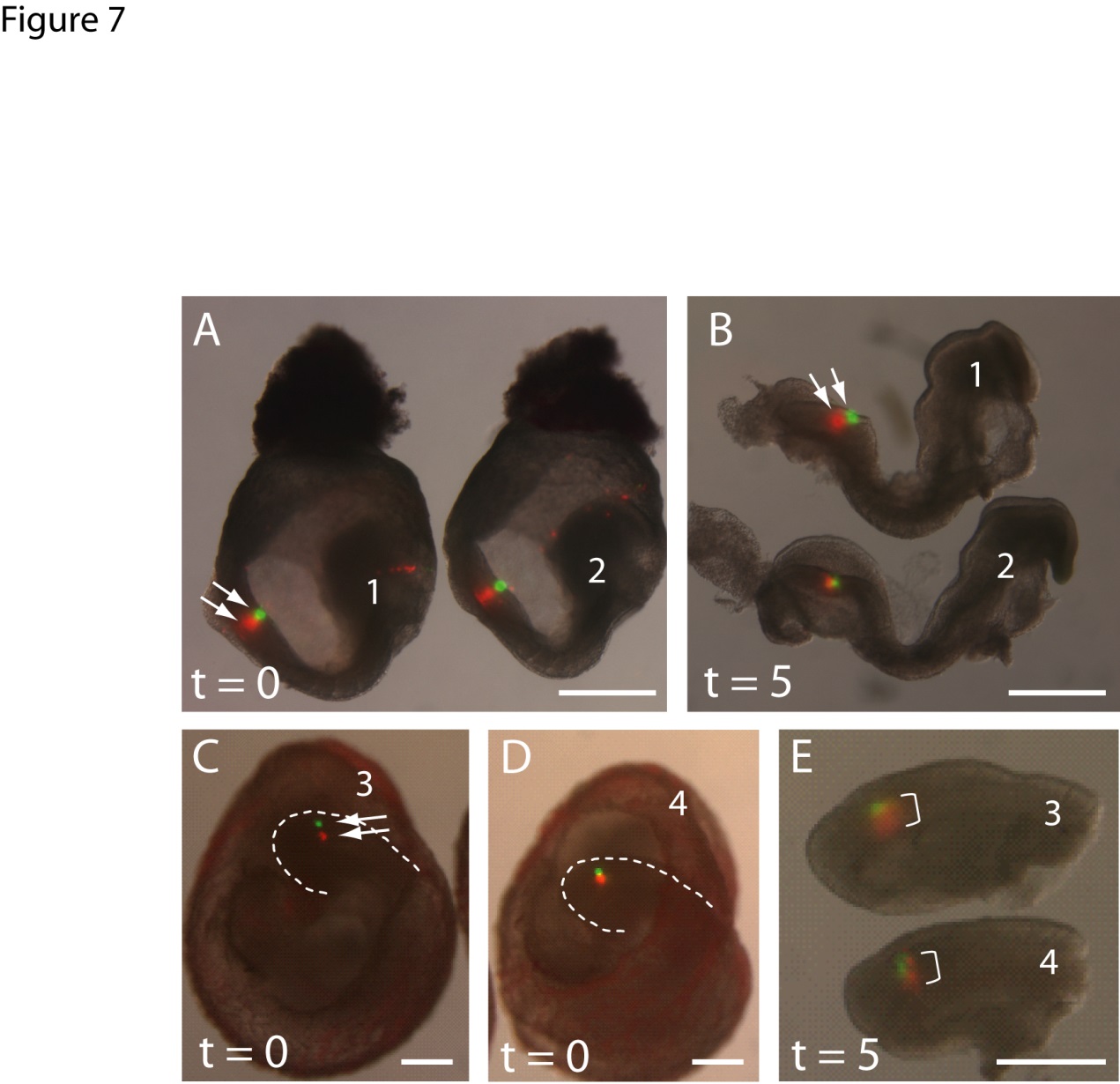


**Figure S2. Short-term vital cell labelling of the neuroepithelium.** DiO (green) and DiI (red) marks in membrane-enclosed embryos pre-culture (t = 0; A, C, D) and in dissected embryos following culture (t = 5 h; B, E). (**A, B**) Two E8.5 embryos (#1, 2) with non-overlapping DiO and DiI marks in the PNP (arrows) at t = 0 (A) and the same two embryos at t = 5 (B). No detectable merging of the signals is seen after culture, at Mode 1 when DLHPs are absent. In this experiment, 10/11 embryos showed no overlap of DiO and DiI marks after culture, while 1/11 showed partial overlap. (**C-E**) Two E9.5 embryos (# 3, 4) with distinct DiO and DiI marks (arrows in C) in the PNP at t = 0 (C, D). Dotted lines outline the caudal region in these membrane-enclosed embryos. Following culture and isolation of the caudal regions (E), a striking increase in dorso-ventral extent of the DiI marks is visible in these embryos. The DiO and DiI marks overlap and partially merge (bracket in E), consistent with ventro-dorsal translocation of DiI-labelled cells at Mode 2 when DLHPs are present. In this experiment, 6/13 embryos showed complete overlap of DiO and DiI marks, 3/13 showed partial overlap and 4/13 showed no overlap after 5 h culture. Scale bars: 0.5 mm.


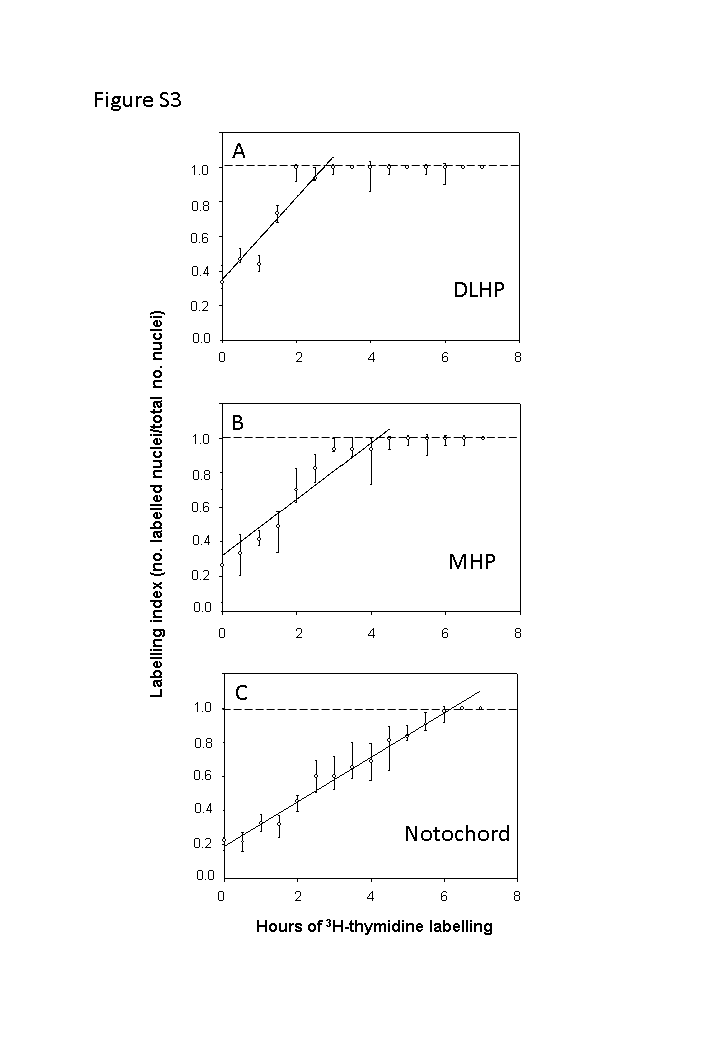


**Figure S3. Change in labelling index with time of exposure to ^3^H-thymidine in whole embryo culture.** Embryos were cultured in the continuous presence of ^3^H-thymidine, and were fixed and sectioned at varying times up to 7 h of culture. Labelling index (LI) was calculated as number of labelled nuclei in the DHLP (A), MHP (B) or notochord (C) divided by the total nuclear number in that tissue/region. LI increased progressively until all cells were labelled in each tissue. Note the major differences between tissues in the slope of the regression line. According to Nowakowski et al (1989), the time taken for LI to reach a plateau is equivalent to the length of G2 + M + G1, while the length of S-phase (Ts) is calculated as y_0_/a, where a is the slope of the regression line and y_0_ is the intercept on the y-axis. These values were used to generate the cell cycle length data shown in Figure 6C.
